# Molecular diversity and functional variability of environmental isolates of *Bacillus* species

**DOI:** 10.1186/2193-1801-3-312

**Published:** 2014-06-25

**Authors:** Ajay Kumar, Amit Kumar, Amit Pratush

**Affiliations:** Department of Microbiology, Shoolini Institute of Life Sciences and Business Management, Solan, Himachal Pradesh 173212 India; School of Biotechnology, Shoolini University, Solan, Himachal Pradesh 173212 India; Department of Biotechnology, Shoolini Institute of Life Sciences and Business Management, Solan, Himachal Pradesh 173212 India

**Keywords:** ACC deaminase, *Bacillus* genetic diversity, Plant growth promoting activity, Rep PCR fingerprinting, 16S rDNA, Seed germination

## Abstract

In the present study, out of 264 phosphate (P) solubilizing *Bacillus* strains isolated from apple rhizosphere, only twelve isolates were found to be efficient (showed most of the plant growth promoting activity) which were further characterized at molecular level using 16S rDNA partial gene sequencing. Out of 12 isolates, MZPSB 207 was found to be most efficient P-solubilizing (864.71 μg/ml) isolate which also showed indole acetic acid production (51.83 μg/ml), siderophore production, ammonia production, antagonistic property (against *Rhizoctonia solani* and *Fusarium oxysporum*), hydrolytic enzymes productions (protease, chitinase and cellulase), 1-aminocyclopropane-1-carboxylate (ACC) deaminase production (7.7 μm αKB mg^-1^ h^-1^). The *in-vitro* seed germination assay showed that *Bacillus* (twelve isolates) inoculated seeds showed more seed germination and seedling vigor rate as compared to uninoculated control treatment.

For the genetic diversity studies of efficient 12 strains, the polyphasic approach using 16S-rDNA, Repetitive element sequence (rep) based PCR (ERIC-PCR and BOX-PCR) were used. Based on 16S rDNA partial gene sequencing the isolated *Bacillus* genus was divide into four groups. First group (five isolates), second group (two isolates), third group (three isolates) and fourth group (two isolates) which showed close genetic relatedness to the *B. subtilis*, *B. pumulis*, *B. megaterium* and *B. amyloliquefaciens*, respectively. The rep PCR fingerprinting showed variability between and within the species. The large variability was showed by ERIC-PCR whereas some variability was showed by BOX-PCR. The results clearly showed that 16S rRNA gene sequencing is unable to discriminate the isolates at strain level. But rep-PCR fingerprinting is excellent tool to characterize and discriminate the strains at the genomic level.

## Background

*Bacillus* is one of the genetically diverse, spore forming, gram positive bacteria (Bhandari et al. 2013). It is widely distributed in various ecological niches and commonly isolated strain. This genus has wide applications.

*Bacillus* has variety of roles in ecology, biotechnology, industry as well as in clinical microbiology, so the various genetic diversity studies on this particular genus have been made. Some species shows same morphological and biochemical features due to this it is still very difficult to characterize new isolates (Harrel et al. 1995), which makes it hard to separate them. Beside this, the environmental isolates of *Bacillus* showed variability in its physiological, nutritional requirements and its genetic content. Increasingly molecular techniques are used for quick species identification. For comparative study and to discriminate the genomes of bacteria various molecular techniques are used like 16S rRNA gene sequencing, Repetitive element sequence-based PCR (rep-PCR). The rep-PCR fingerprinting is found to be very useful molecular technique to discriminate between the species, as this fingerprinting use various DNA segments present in the bacterial genome (Ishii and Sadowsky 2009).

*Bacillus* isolates originating from different sources have been studied, but in the rhizosphere huge microbial diversity is present. The bacteria in the rhizosphere help in the plant growth by using various mechanisms and that is why these bacteria are known as plant growth-promoting rhizobacteria (PGPR) (Farina et al. 2012). Phosphorus is an essential mineral nutrient that often limits plant growth because of its low solubility and fixation in the soil. Under poor available phosphorus soils, phosphate-solubilizing microorganisms play an important role in solubilization of insoluble phosphates and make it available to plants in soluble form which ultimately increases the plant growth (Tripura et al. 2007).

Due to spore forming capability *Bacillus* is one of the most important genera among PGPR which help in the formation of stable bioinocluant. Various environmental factors generate stress that affects the activity or potential of the local micro-flora (Vriezen et al. 2006). Therefore, study of phosphate-solubilizing *Bacillus* under stress conditions from the rhizosphere is the key area to screen new strains that has immense potential to be used as bioinoculant in the agriculture. *Bacillus* species are known already to be as phosphate solubilizers but scanty of reports are available on the potential of phosphate solubilizer under stress conditions. As well as it is well documented in the literature that changing in the ecological niche or geographical region there is definite genetic variability at genus level, species level etc. But, it is interesting to study whether there is genetic variability occurs at genus level or species level from the same ecological niche or not? Therefore, present study was designed to explore the culturable *Bacillus* diversity in the rhizosphere of apple with the help of molecular tools and to characterize these isolates with respect to plant growth promoting traits.

## Results and discussion

### Isolation and screening of P-solubilizer Bacillus isolates

In the rhizosphere root exudation play an important role in defining the functional communities and diversity which varies with plant species. In the rhizosphere these microbial communities have beneficial, neutral or detrimental interaction with plant roots and therefore influence the plant growth (Bakker et al. 2013; Zelicourt et al. 2013). From the huge diversity in the rhizosphere, *Bacillus* is frequently isolated from rhizosphere which generally influences the plant growth positively.

After nitrogen, phosphorus is second major nutrient required for the plant growth. Generally, plenty of phosphorus is available in the soil but it is in the fixed form (insoluble mineral form). So, to overcome the deficiency of phosphorus, farmers generally used phosphatic fertilizers but unfortunately most of the applied fertilizer is fixed in the soil in the form of Al/Fe phosphate in acidic soils and as a calcium phosphate in the alkaline soils (Bashan et al. 2013; Sharma et al. 2013). Therefore, there is need to convert this non-available form of phosphorus to the available form by some cheap and ecological sound method. Under such condition phosphate (P) solubilizers play an important role in providing usable form of P to the plants without affecting the soil health.

Keeping all this in view, a total of 264 phosphate solubilizing *Bacillus* strains were isolated in the present study from *Malus domestica* rhizosphere which showed clear halo zones around the colony. The size of zone of solubilization varies between 3 to 29 mm on Pikovskaya (PVK) agar medium containing TCP (tricalcium phospahte). Out of 264 isolates only twelve were found to show clear and greater than 10 mm zone of solubilization. So, these 12 isolates were selected for further study. As earlier literature shows that TCP alone is not excellent criterion for the selection of P-solubilizer as the isolate may or may not shows P-solubilization on Al/Fe phosphate or calcium phosphate (hydroxyapatite, brushite etc.) therefore, these 12 isolates were tested for P-solubilization in a plate containing Al-P, Fe-P and hydroxyapatite separately. Except for isolates MBPSB5, MBPSB37 and MBPSB69, all other isolates showed zone of solubilization on three tested insoluble phosphate source but it was less as compared to TCP. It was also observed that zone of solubilization in hydroxyapatite plates were less as compared to Al-P and Fe-P.

As the direct measurement of phosphate solubilization in broth assay is likely to give more reliable results than a regular plate assay, the screened 12 phosphate-solubilizing strains were further tested for their ability to solubilize TCP in NBRIP broth (Table  1). The seven isolates showed maximum solubilization on the 7^th^ day of incubation and their maximum values of P solubilized varied from 263.71 to 825.03 μg P/ml (Table  1). Five isolates showed maximum solubilization on 5^th^ day of incubation and their values varied from 221.63 μg P/ml to 864.71 μg P/ml. After reaching maximum value of solubilization, in most of the isolates (irrespective of the day of maximum solubilization) the solubilization decreased thereafter and continued upto 11^th^ day. The isolate MBPSB 207 was found to be most efficient phosphate solubilizer which showed 864.71 μg P/ml of phosphate solubilization which was statistically higher than the other tested isolates. In case of other source of phosphorus (Al-P, Fe-P and hydroxyapatite) tested, there was less P-solubilization as compared to TCP with maximum activity of 529.47 μg P/ml in Fe-P, 396.58 μg P/ml in Al-P and 142.73 μg P/ml in Ca-P was showed by the isolate MBPSB 207. The reason for this may be the low solubility of these stable minerals phosphates as compared to the TCP (Sulbaran et al. 2009; Bashan et al. 2013).

**Table 1:** **Quantitative assay of phosphate solubilization and pH changes exhibited by different** ***Bacillus*** **isolates in NBRIP broth**

	Phosphate solubilization (μg/ml)					pH of medium				
	Days of incubation					Days of incubation				
Isolate	3	5	7	11	Mean	3	5	7	11	Mean
MBPSB 5	262.76	411.69	508.41	358.36	385.30	6.71	5.48	5.35	5.03	5.64
MBPSB 12	341.69	572.02	416.38	341.22	417.83	6.19	5.62	4.93	5.38	5.53
MBPSB 29	329.41	607.53	738.02	585.92	565.22	6.52	5.07	4.51	3.89	4.99
MBPSB 37	194.17	297.52	481.27	313.52	321.62	6.28	5.43	5.72	4.96	5.60
MBPSB 69	157.34	221.63	263.71	198.37	210.26	5.84	4.96	4.21	3.92	4.73
MBPSB 124	398.25	806.12	664.49	407.36	569.05	6.58	5.54	5.76	6.15	6.00
MBPSB 147	291.47	572.59	825.03	576.35	566.36	6.47	5.35	4.69	4.84	5.34
MBPSB 164	236.43	519.78	452.64	305.72	378.64	5.92	5.19	5.32	4.97	5.35
MBPSB 194	216.79	380.03	647.84	473.16	429.45	6.03	6.12	5.44	4.89	5.62
MBPSB 207	426.37	864.71	725.61	591.04	651.93	6.39	5.21	4.85	4.21	5.41
MBPSB 219	204.23	415.85	613.37	483.51	429.24	6.14	5.05	5.57	4.73	5.37
MBPSB 259	424.74	529.16	437.58	262.03	367.88	5.88	5.07	4.63	4.85	5.11
Mean	275.13	516.55	564.53	408.05		6.24	5.34	5.08	4.82	
Variant	SEm±	CD (P ≤ 0.01)				SEm±	CD (P ≤ 0.01)			
Isolate	0.214	0.937				0.064	0.273			
Day	0.081	0.348				0.026	0.091			
Interaction	0.526	1.563				0.159	0.384			

After reaching maximum value of solubilization, in most of the isolates (irrespective of the day of maximum solubilization) the solubilization decreased thereafter and continued upto 13^th^ day. The reason for this trend may be attributed to the fact that when the rate of uptake is higher than that of solubilization, a decrease in P concentration in the medium could be observed. On the contrary, when the uptake rate decreases, the level of P in the medium increases (Rodriguez and Fraga 1999).

The pH of the growth medium changed during the process of solubilization from its initial value of 7.0 to 3.89 in majority of the isolates. The isolate MBPSB 207 showed a maximum solubilization of 864.71 μg P/ml at pH of 5.21 on 5^th^ day of incubation. In the present study, no relationship could be ascertained with the quantity of P-solubilized and value of pH.

### Screening of isolates for plant growth promoting activities

One of the direct mechanisms by which PGPR promote plant growth is by the production of phytohormones as well as siderophores (Glick 1995). Out of 12 strains of phosphate solubilizer, ten, eleven and twelve isolates were found to produce siderophore, ammonia and indole-acetic acid (IAA), respectively (Table  2). MBPSB 207, the most efficient isolate with respect to phosphate solubilizing activity, showed 51.83 μg/ml of IAA production which was significantly higher than the other strains except for isolate MBPSB 37. Auxins (IAA and derivatives) are responsible for division, extension, and differentiation of plant cells and tissues (Mano and Nemoto 2012; Duca et al. 2014). In microorganisms various known pathways of IAA biosynthesis are find out which may be tryptophan dependent or independent (Tsavkelova et al. 2006; Duca et al. 2014). In the present study medium was amended with tryptophan (5 mM) which act as a precursor for the biosynthesis of IAA. In natural condition the tryptophan may be available in the rhizosphere through root exudates as noticed by the Beniziri et al. ( 1998).

**Table 2:** ***Bacillus*** **isolates showed various plant growth promoting traits**

Isolate	IAA (μg/ml)	Siderophore	Ammonia	Protease	Chitinase	Cellulase	ACC deaminase (μm αKB mg ^-1^ h ^-1^)	Antifungal activity	
								***Rhizoctonia solani***	***Fusarium oxysporum***
MBPSB 5	17.12 ± 1.9	++	+++	+	++	++	5.8 ± 0.5	++	+
MBPSB 12	21.94 ± 2.1	++	+	+	++	-	8.1 ± 0.8	+	+++
MBPSB 29	19.36 ± 2.0	++	+	+	+++	-	1.5 ± 0.1	-	-
MBPSB 37	53.61 ± 4.7	+	++	-	+	++	4.8 ± 0.3	+	+
MBPSB 69	27.47 ± 2.8	-	+++	+	+	++	8.6 ± 0.7	+	++
MBPSB 124	35.68 ± 3.3	+	++	+	+	-	7.4 ± 0.5	+	+
MBPSB 147	15.72 ± 1.8	++	++	+++	-	-	6.5 ± 0.4	+++	++
MBPSB 164	31.97 ± 3.0	+++	-	-	-	+	-	-	-
MBPSB 194	46.84 ± 4.1	+	++	-	++	++	5.2 ± 0.5	++	+
MBPSB 207	51.83 ± 4.2	++	+++	++	++	+++	7.7 ± 0.6	+++	+++
MBPSB 219	22.39 ± 2.1	-	++	+	+	-	1.1 ± 0.1	+	+
MBPSB 259	38.73 ± 3.6	+++	+	++	+	+++	0.7 ± 0.04	+	+

The isolate MBPSB 207 was also found positive for siderophore and ammonia production*.* Siderophores are low molecular weight iron chelating ligands synthesized by microorganisms. Microbial siderophore may stimulate plant growth directly by increasing the availability of iron in the soil surrounding the roots or indirectly by competitively inhibiting the growth of plant pathogens by scavenging iron and making it less available to the pathogens (Fgaier and Eberl 2011). Similarly, ammonia is considered as one of the plant growth promoting metabolites and some authors considered the production of ammonia to be involved in antagonistic interaction that results in disease control (Saraf et al. 2011). Nevertheless, meticulous experimentation is required to exactly pin point the role of ammonia in plant growth and disease suppression.

Beside biostimulators, some PGPR are also reported to act as a bioprotectants and inhibit the fungal growth therefore in the present study 12 efficient isolates were tested for antifungal activity (Table  2). Twelve isolates except for isolate MBPSB 29 and MBPSB 164, were found to inhibit the growth of phytopathogenic fungus i.e. *Fusarium oxysporum* and *Rhizoctonia solani*. The most efficient isolate MBPSB 207 with respect to phosphate solubilization, showed more zone of inhibition as compared to the other isolates. Further, these twelve isolates were tested for the production of hydrolytic enzymes (Table  2). These enzymes are thought to play a role in antifungal activity (Krakova et al. 2012). Out of twelve isolates 9, 10 and 7 strains were showed protease, chitinase and cellulase production, respectively. The strains MBPSB 147 showed highest protease activity, isolate MBPSB 29 showed highest chitinase activity whereas, isolates MBPSB 207 and MBPSB 259 showed highest cellulose activity. The isolate MBPSB 207 was found to show all the three enzyme activities. Beside these hydrolytic enzymes as antifungal molecules, IAA, siderophores and ammonia are also thought to involve in antifungal activity. IAA in combination with glutathione-s-transferases inhibits the spore germination and filament growth of fungus (Hahn and Strittmatter 1994). Whereas, siderophores indirectly showed antifungal activity by competitively inhibiting the growth of plant pathogens by scavenging iron and making it less available to the pathogens (Fgaier and Eberl 2011). The production of ammonia involved in antagonistic interaction and therefore acts as bioprotectant (Saraf et al. 2011) however, meticulous experimentation is required to exactly pin point the role of ammonia in suppressing the diseases.

Another important attribute as a PGPR is the production of ACC deaminase enzyme which lowers the ethylene levels and thus helps in the germination of seed and elongation of root (Belimov et al. 2001, Glick 2014). In the present work the highest ACC deaminase activity (Table  2) was showed by isolate MBPSB 69 (8.6 μm αKB mg^-1^ h^-1^) which was at par with isolate MBPSB 12 (8.1 μm αKB mg^-1^ h^-1^). The most efficient isolate MBPSB 207 showed 7.7 μm αKB mg^-1^ h^-1^ ACC deaminase activity, which was significantly lower than isolate MBPSB 69.

The isolate MBPSB 207 yielded a 729-bp DNA fragment by use of the specific primer pair. Gene sequencing and BLASTN analysis confirmed the presence of *acdS* gene in representative MBPSB 207 strain associated with *Malus* tree. The *acdS* gene of isolate MBPSB 207 showed 96% similarity with *Klebsiella pneumoniae* (JN625725), followed by 96% similarity with *Pseudomonas putida* (HM053973). The occurrence and expression of *acdS* gene in this isolate suggested that this isolate possess the potential for improving plant growth.

### Seed germination assay

**Figure 1:**
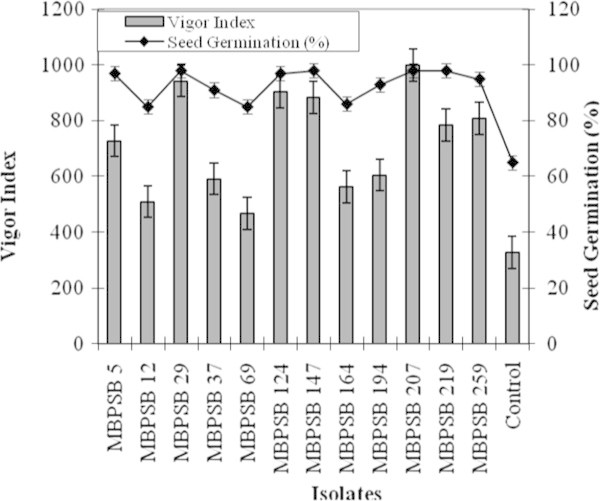
**Seed germination and vigor index of maize seeds inoculated with efficient phosphate solubilizing bacterial isolates.**

The seeds are the reproductive units which are expected to give rise the healthy plant. During seed germination there are many morphological and physiological changes occurs, which activate the embryo and many factors influences the seed germination like temperature, moisture, hormones, enzymes etc. (Miransari and Smith 2014). In the present study, the effect of 12 efficient P-solubilizing strains on seed germination and vigor index is shown in Figure  1. The results clearly showed that the highest seed vigor index was noticed in case of isolate MBPSB 207 (998) followed by the isolate MBPSB 29 (941). All the isolates showed higher seed germination rate as compared to the control (uninoculated seeds). The highest seed germination rate (98%) was showed by four isolates i.e. MBPSB 29, MBPSB 147, MBPSB 207 and MBPSB 219. These results were similar to the research report as given by the earlier worker (Mia et al. 2012). The increased rate of seed germination and seedling vigor in PGPR treated seeds as compared to control may be due to the release of various growth hormones like IAA, cytokinins, gibberellins etc. (Hayat et al. 2010). The gibberellins are involved in inducing the genes which help in the secretion of various enzymes like protease, nuclease, and hydrolases especially α-amylase which are involved in assimilation and hydrolysis of starch that ultimately leads to higher seed germination rate (Gholami et al. 2009; Miransari and Smith 2014). The rate of seed germination also influenced by IAA which interact and cross talk with gibberellins and ethylene. Also IAA up regulates the enzyme glyoxalase I which enhance the cell growth and development (Miransari and Smith 2014). Cytokinins help in increase seed germination by helping in to overcome the stresses like draught, salinity, oxidative etc. (Peleg and Blumwald 2011; Miransari and Smith 2014).

### Biochemical and molecular characterization

The 12 efficient strains were characterized on the basis of their morphological and biochemical characteristics (Table  3). All the strains were gram positive, rods, motile, hydrolysis starch except for strains MBPSB 12 and MBPSB 147. Only one strain (MBPSB 194) was able to produce H_2_S. The other biochemical parameters are presented in Table  3.

**Table 3:** **Characterization of** ***Bacillus*** **strains obtained from the apple rhizosphere**

***Bacillus***strains												
Characteristics	MBPSB 5	MBPSB 12	MBPSB 29	MBPSB 37	MBPSB 69	MBPSB 124	MBPSB 147	MBPSB 164	MBPSB 194	MBPSB 207	MBPSB 219	MBPSB 259
**Endospore**	**+**	**+**	**+**	**+**	**+**	**+**	**+**	**+**	**+**	**+**	**+**	**+**
**Colony color**	White	White	White	White	White	Off-white	White	White	White	White	White	White
**Pigmentation**	-	-	-	-	-	-	-	-	-	-	-	-
**Motility**	+	+	+	+	+	+	+	+	+	+	+	+
**Starch hydrolysis**	+	-	+	+	+	+	+	+	+	+	+	+
**Gelatine hydrolysis**	+	+	+	+	+	+	+	+	+	+	+	+
**H**_**2**_ **S production**	-	-	-	-	-	-	-	-	+	-	-	-
**Glucose fermentation**	+	+	+	+	+	+	+	+	+	+	+	+
***Biochemical parameters***												
**Catalase**	+	+	+	+	+	+	+	+	+	+	+	+
**Oxidase**	+	+	+	+	+	+	+	+	+	+	+	+
**Indol**	-	-	-	+	-	-	-	-	-	-	-	+
**VP test**	+	+	+	-	-	-	+	+	-	+	-	+
**Citrate**	+	+	+	+	+	+	+	+	-	-	+	-
**Nitrate reduction**	-	-	+	+	+	-	-	+	-	+	-	+
**Urease**	-	+	-	+	+	+	+	-	-	+	+	+

**Figure 2:**
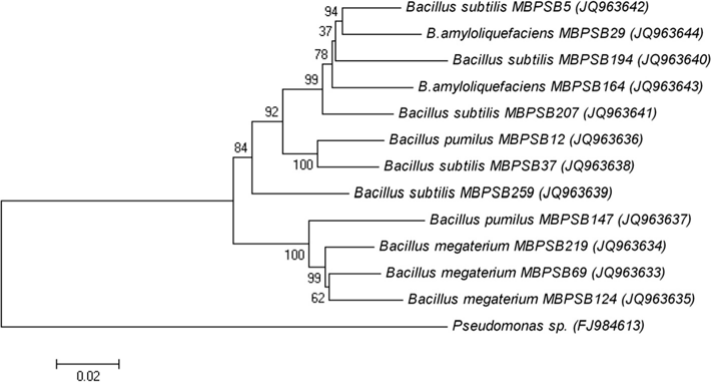
**Phylogenetic tree of indigenous members of the genus** ***Bacillus*** **, based on 16S rRNA gene sequences.** (Scale bar, 0.02 substitutions per nucleotide position).

To further confirm the results of biochemical analysis, the 16S rDNA gene sequence analysis was carried out. Their phylogenetic allocation and 16S rRNA gene sequence identities are presented in Figure  2. All the strains according to similarity values (98%) indicated that these all belong to genus *Bacillus* viz. *B. amyloliquefaciens* (MBPSB 29 and MBPSB 164), *B. megaterium* (MBPSB 69, MBPSB124 and MBPSB 219), *B. pumilus* (MBPSB 12 and MBPSB 147) and *B. subtilis* (MBPSB 5, MBPSB 37, MBPSB 194, MBPSB 207 and MBPSB 259).

**Figure 3:**
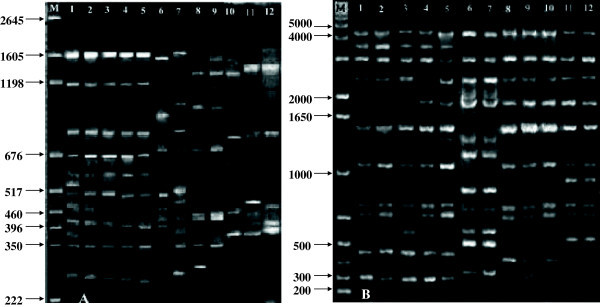
**DNA fingerprinting patterns of indigenous isolates generated by (A) ERIC-PCR (B) BOX-PCR.**

Since, the efficiency of 16S rRNA gene sequences are often limited for the identification of bacteria at species or strain levels, therefore DNA fingerprint methods have been developed to characterize and discriminate *Bacillus* strains (Weisburg et al. 1991; Janda and Abbott 2007). Therefore, to distinguish variability between and within bacterial strains a polyphasic genotypic fingerprinting tools i.e. ERIC and BOX-PCR were used in the present work (Figure  3). A number of bands were generated in the present study by all the twelve strains using ERIC and BOX-PCR which shows polymorphism in band pattern [Figure  3A and 3B (Lane 1: MBPSB 5, Lane 2: MBPSB 37, Lane3: MBPSB 194, Lane 4: MBPSB 207, Lane 5: MBPSB 259, Lane 6: MBPSB 12, Lane 7: MBPSB 147, Lane 8: MBPSB 29, Lane 9: MBPSB 164, Lane 10: MBPSB 69, Lane 11: MBPSB 124 and Lane 12: MBPSB 219 )]. One hundred and thirty three bands were observed by BOX-PCR (300-4200 bp) and 99 bands by ERIC-PCR (292-1605 bp). Bands pattern generated by ERIC-PCR showed large variability in all the tested 12 isolates as shown in Figure  3. Similarly, bands generated by BOX-PCR were also showed some variability for example in *B. subtilis* group except for isolate MBPSB 194 all the other four isolates (MBPSB 5, MBPSB 37, MBPSB 207 and MBPSB 259) showed similar bands pattern (Figure  3). In case of *B. megaterium* group (MBPSB 69, MBPSB124 and MBPSB 219) isolate MBPSB124 showed different band pattern. Whereas, *B. pumilus* group (MBPSB 12 and MBPSB 147) and *B. amyloliquefaciens* group (MBPSB 29 and MBPSB 164) showed same bands pattern.

The characteristic feature of repetitive sequences (ERIC and BOX) is that they contain repetitive, non-coding sequences which are distributed through out the genome in a unique fashion and that help in discriminating the bacteria (Chudzik and Stosik 2005). The BOX element (154 bp) have three subunits i.e. BOX A, BOX B and BOX C of which BOX A is more conserved, whereas ERIC sequence (124-127 bp) has central, conserved palindromic sequence and therefore these conserved parts are used as a target in molecular biology for discrimination of bacterial species (Versalovic et al. 1994; Rademaker et al. 2005). On amplification of these repetitive sequences they yield a unique band patterns that serve as unique identifier (Versalovic et al. 1998). Also, these polyphasic genotypic fingerprinting techniques are excellent and reliable tools as compared to 16S rRNA gene sequences for discriminating the environmental *Bacillus* isolates as a separate group (Kim et al. 2003; Patil et al. 2010). The results clearly showed that 16S rRNA gene sequencing is unable to discriminate the isolates at strain level. But rep-PCR fingerprinting (ERIC and BOX-PCR) is excellent and reliable tools to characterize isolates at the genomic level.

### P-solubilization under stress conditions

The stressful conditions prevailing in the soil have direct effect on the survival and proliferation of the soil microbiota (Miller and Woods 1996; Zahran 1999; Fierer and Jackson 2006). The most efficient isolate MBPSB 207 was further tested for phosphate (P) solubilization under stress conditions. As evident from Table 4 that at 5^th^ day of incubation, the highest P-solubilization was observed at temperature 37°C (831.72 μg/ml) which was significantly higher than the other tested stress followed by pH 6.5 (817.38 μg/ml). The highest reduction in P solubilization was occurred at temperature of 15°C (583.63 μg/ml), followed by pH 9 (597.01 μg/ml) at 5^th^ day of incubation. Overall, the isolate MBPSB 207 survived well under different stress conditions and solubilized insoluble phosphorus. The high stress tolerance capacity of the tested isolate to different stress conditions indicated that biochemical and molecular system help the bacteria to adapt to these stressful conditions. The screening of stress-tolerant strain could be an important attribute in selection of indigenous strains for developing microbial inoculants to be used as biofertilizer for sustainable agriculture.

**Table 4:** **P-solubilization by most efficient isolate (MBPSB 207) under different stress conditions**

P-solubilization (μg/ml)					
Stress	Days of incubation				Mean
	3	5	7	11	
**NaCl 3.5%**	298.77	623.33	524.88	417.86	466.21
**NaCl 7%**	302.53	712.49	602.97	514.67	533.17
**pH 5.5**	324.71	783.46	563.62	459.52	532.83
**pH 6.5**	391.55	817.38	616.35	524.51	587.45
**pH 7.5**	339.64	746.29	509.82	442.6	509.59
**pH 9**	216.18	597.01	412.43	305.40	382.75
**Tem 15°C**	302.58	583.63	463.90	375.52	431.41
**Temp 37°C**	395.41	831.72	661.49	518.61	601.81
**Mean**	321.42	711.91	544.43	444.84	
**Variants**	SEm±	CD (p ≤ 0.01)			
**Days**	0.35	1.72			
**Stress**	0.5	2.44			
**Interactions**	1	4.88			

## Conclusions

In summary, the present study have generated very useful information regarding genetic variability in plant growth promoting *Bacillus* strains which are also tolerant to various stress like temperature, pH and salinity. These results are very informative regarding development of bioinoculants for sustainable agriculture especially for increasing maize crop production and also this isolate (MBPSB 207) work efficiently under different stress conditions. But, further studies are required to check the potential and performance of these isolates under different field conditions. The studied isolates were categorized into four groups: *B. amyloliquefaciens*, *B. megaterium*, *B. pumilus* and *B. subtilis* depending on molecular characterization. Even though polyphasic technique was used to identify the *Bacillus* isolates but this approach was unable to discriminate the isolates at species level. Although, these strains were isolated from the same environmental source but they showed heterogeneity in their phenotypic and genetic characters.

## Methods

### Sampling and bacterial isolates

Soil samples were collected from the rhizosphere of *Malus domestica* (Apple) growing in orchid at Theog, Shimla (India) at an elevation of about 2397 m above mean sea level and soil samples were heated at 80 ± 1°C for 10 min before use. Tryptic soy agar was used for the isolation of bacterial strains by serial soil dilution technique and plates were incubated at 30 ± 1°C. The colonies obtained were purified by restreaking and stocked for further studies.

### Screening of efficient phosphate-solubilizing isolates

The bacterial isolates were spot inoculated on PVK agar plates and the halo zones formed around the colony was measured after regular interval of times at 30 ± 1°C). For quantitative estimation of phosphate solubilization, NBRIP broth (containing 0.5% TCP) was inoculated with screened isolates as described earlier by Nautiyal ( 1999) and vanado-molybdate method was used for the estimation of solubilized phosphorus (Jackson 1973). Alternative to TCP, iron phosphate, aluminium phosphate and hydroxyapatite were also tested at the rate of 0.5% for qualitative and quantitative analysis of most efficient isolates screened on TCP as described above.

### PGP characteristics of the bacteria

#### Indole-3-acetic acid (IAA) production

For IAA production assay, bacterial cultures were grown until stationary phase in Luria-Bartani medium (LB) supplemented with 5 mM L-tryptophan (Sigma) at 30 ± 1°C in an orbital shaker at 100 rpm. Cell free supernatant was used to estimate the IAA by following the method of Gordon and Weber ( 1951).

#### Siderophore production

The agar plates containing Chrom Azurol S (CAS) dye was used for the detection of siderophore production (Schwyn and Neilands 1987). Colonies showing orange halos around them were considered as siderophore producer.

#### Ammonia production

For the detection of ammonia production organism was grown in 5 ml of peptone water for 48-72 hrs at 30 ± 1°C. After incubation 1 ml of Nessler’s reagent was added to inoculated peptone water and development of faint yellow to dark brown color indicate the production of ammonia (Bakker and Schippers 1987).

#### Screening of bacterial isolates for antagonistic activity

To determine antagonistic activity of the isolates against fungal pathogens *Rhizoctonia solani* and *Fusarium oxysporum* (obtained from Dept. of Microbiology, CSK HPKV, Palampur, India), dual culture technique was used. For this, 96 hrs old culture of fungus was used. Agar block of 5 mm diameter was spot inoculated at the centre of the agar plate and tested bacterial isolate was spot inoculated 2 cm away from the fungal culture. The plates were incubated at 30 ± 1°C for 96 hrs and checked for the antagonistic activity after 96 hrs.

#### Enzyme tests

(i) Protease
  The screened isolates were spot inoculated on skim milk agar and plates were incubated at 30 ± 1°C for 96 hrs. The isolate showing clear zone around the colony was considered as protease producer.
(ii) Chitinase
  The chitin agar medium plates were spot inoculated with log phase screened *Bacillus* cultures and plates were incubated at 30° ± 1°C for 96 hrs. The clear zone around the colony was considered as chitinase producer.
(iii) Cellulase
  The cellulolytic activity was determined on Carboxymethyl cellulose (CMC) agar medium containing 1% CMC as described earlier by Ariffin et al. (2006).

#### Assay of 1-aminocyclopropane-1-carboxylate deaminase (ACCD)

In different samples the absorbance of α-ketobutyrate generated by the hydrolysis of 1-aminocyclopropane-1-carboxylate (ACC) by the enzyme ACC deaminase in the cell free extract was compared at 540 nm by using α-ketobutyrate as a standard ranging between 0.1 and 1.0 μmol (Saleh and Glick 2001; Penrose and Glick 2003). For ACCD activity bacteria was grown, centrifuged at 8000 rpm and cell pellet was washed with Dworkin and Foster (DF) mineral medium and resuspended in 7.5 ml of DF containing 3 mM ACC. The inoculated DF medium was incubated at 30 ± 1°C for one day, centrifuged and resuspended in 1 ml of 0.1 M Tris–HCl buffer (pH 7.6) and centrifuged at 8000 rpm for 15 minutes. Then 600 μl of 0.1 M Tris–HCl buffer (pH 8.5) was added to the pellet, vortex and finally 30 μl of toluene was added. The quantity of α-ketobutyrate produced in the cell free suspension was used for the quantification. The solution containing no cell suspension or no ACC were used as controls.

### Seed germination bioassay

*In-vitro* seed germination assay was conducted using twelve efficient PGPR isolates by soft agar plate method on *Zea mays* (maize) seeds. The bacterial cultures were grown in Tryptic soy broth, centrifuged, washed with sterilized phosphate buffer saline (PBS) and finally suspended in the sterilized PBS until the population reaches to 10^8^ cells per ml. Before *in-vitro* testing, the surface of seed was sterilized as described earlier by Johnston-Monje and Raizada ( 2011). The seeds were dipped in bacterial cultures for 20 minutes and then placed in the soft agar plate containing 0.8% sterilized agar. The plates were incubated at 28 ± 1°C for 3-5 days and seeds were observed for hypocotyl length and root length. By using the following formula the vigor index (VI) was calculated: (mean hypocotyl length + mean root length) x% germination.

### *PCR amplification and sequencing of g*enes *encoding ACC deaminase (acdS genes) in Bacillus*

Partial *acdS* genes were amplified by using primers Deg ACCf (5’-GGBGGVAAYAARMYVMGSAAGCTYGA-3’) and Deg ACCr (5’-TTDCCHKYRTANACBGGRTC-3’) as described by Nikolic et al. ( 2011). PCR amplification was done using reaction mixture containing 20 ng of DNA sample, 20 μM dNTPs, 4 pmol of each primer, 2 mM MgCl_2_ and 1U *Taq* polymerase under following conditions: initial denaturation for 5 min at 95°C, followed by 30 cycles of denaturation for 37 sec at 95°C, annealing for 34 sec at 55°C, and elongation for 1 min at 72°C and then a final 5 min elongation at 72°C. The purified product was sent for custom sequencing.

### Phenotypic characterization of bacterial isolates

Morphological and biochemical characteristics of the efficient bacterial isolates were studied by the methods described in Bergey’s Manual of Systematic Bacteriology (Holt et al. 1994).

### Sequence analysis of 16S rDNA

Genomic DNA of isolates was extracted by using QIAamp DNA Mini Kit (Qiagen, Valencia, CA) by following the manufacturer instructions. The primers used for 16S rDNA amplification were 27 F (5’-AGAGTTTGATCCTGGCTCAG-3’) and 1492R (5’-GGTTACCTTGTTACGACTT-3’) (Meier et al. 2012). The thermo cycling conditions consisted of an initial denaturation step at 94°C for 5 min, followed by 33 cycles of 94°C for 1 min, 52°C for 1 min, and 72°C for 2 min, and final extension at 72°C for 5 min. The gel purified 16S rDNA was sent for custom sequencing. The phylogenetic tree was constructed by MEGA 3.1 using the Neighbor-Joining (N-J) method. The robustness of the phylogenetic tree topology was evaluated with 1000 replicates of bootstrap analysis.

### rep-PCR DNA fingerprinting

The genomic fingerprinting of efficient strains was subjected to two types of rep-PCR using the BOX primer (Versalovic et al. 1994) and ERIC primer (Versalovic et al. 1991). The ERIC primer i.e. ERIC1R (5’-ATGTAAGCTCCTGGGGATTCAC-3’) and ERIC2 (5’-AAGTAAGTGACTGGGGTGAGCG-3’) whereas BOXA1R (5’-CTACGGCAAGGCGACGCTGACG-3’) were used for the amplification cycling of the rep-PCR. The amplified products were resolved by electrophoresis in 1.5% agarose containing ethidium bromide (0.5 μg/ml) for 2.5 h at 80 V. The fingerprints were visually compared.

### Phosphate solubilization under stress conditions

The most efficient bacterial isolate was subjected to various stress parameters like pH (5.5, 6.5, 7.5 and 9.0), temperature (15°C and 37°C) and sodium chloride concentration (3.5% and 7.0%) in NBRIP broth and phosphate solubilized was quantified as discussed earlier. The uninoculated sterilized medium served as control.

### Statistical analysis

The software STATISTICA version 7 (StatSoft® Inc. Tulsa, USA) was used on present study data for the ANOVA and all the experiments were done in triplicates.

### Submission of gene sequences

DNA sequences: The 16S rDNA partial sequences of phosphate-solubilizing *Bacillus* spp. were deposited in the GenBank database under the accession numbers: JQ963633 (MBPSB 69), JQ963634 (MBPSB 219), JQ963635 (MBPSB 124), JQ963636 (MBPSB 12), JQ963637 (MBPSB 147), JQ963638 (MBPSB 37), JQ963639 (MBPSB 259), JQ963640 (MBPSB 194), JQ963641 (MBPSB 207), JQ963642 (MBPSB 5), JQ963643 (MBPSB 164), and JQ963644 (MBPSB 29).

The *acdS* gene partial sequence of most efficient isolate MBPSB 207 was deposited in the GenBank database under accession number JQ995371.
